# PET and CT Image Fusion of Lung Cancer With Siamese Pyramid Fusion Network

**DOI:** 10.3389/fmed.2022.792390

**Published:** 2022-03-31

**Authors:** Ning Xiao, Wanting Yang, Yan Qiang, Juanjuan Zhao, Rui Hao, Jianhong Lian, Shuo Li

**Affiliations:** ^1^College of Information and Computer, Taiyuan University of Technology, Taiyuan, China; ^2^School of Information Management, Shanxi University of Finance and Economics, Taiyuan, China; ^3^Thoracic Surgery, Shanxi Cancer Hospital, Taiyuan, China; ^4^Department of Medical Imaging and Medical Biophysics, Western University, London, ON, Canada

**Keywords:** PET-CT fusion, image quality, siamese neural network, pyramid transform, structural similarity

## Abstract

**Background:**

The fusion of PET metabolic images and CT anatomical images can simultaneously display the metabolic activity and anatomical position, which plays an indispensable role in the staging diagnosis and accurate positioning of lung cancer.

**Methods:**

In order to improve the information of PET-CT fusion image, this article proposes a PET-CT fusion method *via* Siamese Pyramid Fusion Network (SPFN). In this method, feature pyramid transformation is introduced to the siamese convolution neural network to extract multi-scale information of the image. In the design of the objective function, this article considers the nature of image fusion problem, utilizes the image structure similarity as the objective function and introduces L1 regularization to improve the quality of the image.

**Results:**

The effectiveness of the proposed method is verified by more than 700 pairs of PET-CT images and elaborate experimental design. The visual fidelity after fusion reaches 0.350, the information entropy reaches 0.076.

**Conclusion:**

The quantitative and qualitative results proved that the proposed PET-CT fusion method has some advantages. In addition, the results show that PET-CT fusion image can improve the ability of staging diagnosis compared with single modal image.

## 1. Introduction

Medical imaging is a technique and process for obtaining images of a certain part of the human body in a non-invasive manner (1–3). With the continuous development of computer imaging technology, medical imaging has derived multi-modal forms. Common medical images can be divided into Computed Tomography (CT), Positron Emission Tomography (PET), Magnetic Resonance Imaging (MRI), UltraSound, and so on. Medical images of different modalities can reflect disease information from different angles. The correlation and complementarity of image information from different imaging modality can be used to merge different modalities of medical image. Effective fusion can provide doctors with richer diagnosis and treatment information. For lung cancer, the common imaging screening procedures mainly include CT and PET (4, 5). Among them, CT images have the characteristics of short scanning time and clear images, which can provide clear human bone tissue anatomy and lesion images, and are widely used for screening diseases such as chest and abdomen; PET uses short-lived radionuclide metabolites (common fluorodeoxyglucose, FDG) to reflect the metabolic activities in the human body to perform imaging. PET has the advantages of high sensitivity and high specificity. The PET-CT fusion image can simultaneously reflect the pathophysiological changes and morphological structure of the lesion. It has important clinical value for the staging of non-small cell lung cancer, the judgment of the recurrence and metastasis of lung cancer, and the formulation of radiation treatment plans.

Medical image fusion technology is an important application area in the field of information fusion technology. The fused image has more information than the source image, which is suitable for human eyes to distinguish, and can further enrich some details of the image, which can provide more practical information for clinical diagnosis. At present, researchers have proposed many PET-CT fusion methods. The mainstream fusion methods mainly include methods based on multi-scale decomposition and methods based on wavelet transformation (6, 7). For example, a multi-modal medical image fusion method in the non-subsampled wavelet transform domain was proposed in (8). This method first performs non-subsampled wavelet transform on the source image, and then uses Pulse Coupled Neural Network(PCNN) and Max selection fusion rule to analyze high frequency sub-band and low frequency sub-band fusion, this strategy simultaneously solves the two problems of energy preservation and detail extraction in image fusion. An image fusion based on guided filtering (9) used average filtering to obtain two-scale base and detail layers, and determined the two-scale weight of the fusion result according to the saliency map of the image. These fusion methods follow certain fusion rules to process the images point by point when fusing images. Therefore, the quality of the fused image is largely affected by the fusion rules, and the noise resistance effect is not high. During multi-scale decomposition and transformation, some original brightness information of the image is often lost.

In recent years, medical image fusion technology based on convolutional neural network (10–12) has been developed rapidly. With the help of symmetric network structure or co-learning method, it automatically learns the direct mapping between the original image and the fused image, which is different from the multi-scale decomposition method and the wavelet transform method, the convolutional neural network learns to extract features from a large number of images autonomously, and can obtain low-level features and high-level semantic features at the same time. In order to obtain a clearer fusion image, the multi-focus convolutional neural network (11) used a high-quality image patch and its blurred patch as input to automatically learn the coding features of the source image and the target image; A novel image fusion framework, IFCNN (10), was designed, which uses convolutional layers to extract salient image features from input images, selects appropriate fusion rules to fuse the extracted features, and finally obtains the fused image through convolutional layer reconstruction. A multi-layer cascaded fusion network was proposed in (12), this end-to-end deep convolutional neural network can automatically perform feature extraction, feature fusion, and image reconstruction on the fused medical images, and use fast deconvolution to reduce the number of features. The main feature of the convolutional neural network is the invariance of feature translation. It does not require complex fusion rules to obtain high-quality fused images and can also retain the structural information in the original image to the greatest extent. However, the convolutional neural network only takes a single-dimensional picture is used as an input, which lacks the diversity of image scales, and the standard convolution still has the problem of unknowable content (13, 14).

The feature pyramid (15, 16) is a method that can efficiently extract the features of each dimension in the picture. The method of image transformation is used to generate images of various scales. The convolutional neural network model is used to express the characteristics of images of different scales from the low to the top, so as to generate feature maps with stronger content expression ability. In order to consider the multi-scale information of the image, this article introduces the feature pyramid transformation in the traditional convolution, which enhances the content of the original convolution feature on the scale. Specifically, this article proposes a Siamese Pyramid Fusion Network, which implements end-to-end image feature-level fusion by constructing a siamese structure and multi-scale feature modules. The network fusion process includes a multi-scale feature extraction stage and bimodal cross-correlation. The fusion stage and the image reconstruction stage consist of three parts. In particular, the contribution of this article can be summarized in the following three aspects:

- We use a siamese structure composed of a PET encoder and a CT encoder. The two encoders have the same structure and share parameters. This structure can extract the two modal image features of PET and CT separately.
- When extracting image feature maps, we design a multi-scale convolution structure. This structure can not only extract the translation invariance of features in the original image, but also increase the multi-scale features of image.
- When designing the objective function, we propose a novel objective function, which takes the structural similarity between the original image and the fusion image as the backbone, and adds the L1 norm as a regularization term to reduce noise interference.

The rest of the article is organized as follows. The Section 2 describes the proposed algorithm in detail. The Section 3 describes the experiment and results. The discussion is in Section 4 and the conclusion follows in Section 5.

## 2. Method

**Figure 1:**
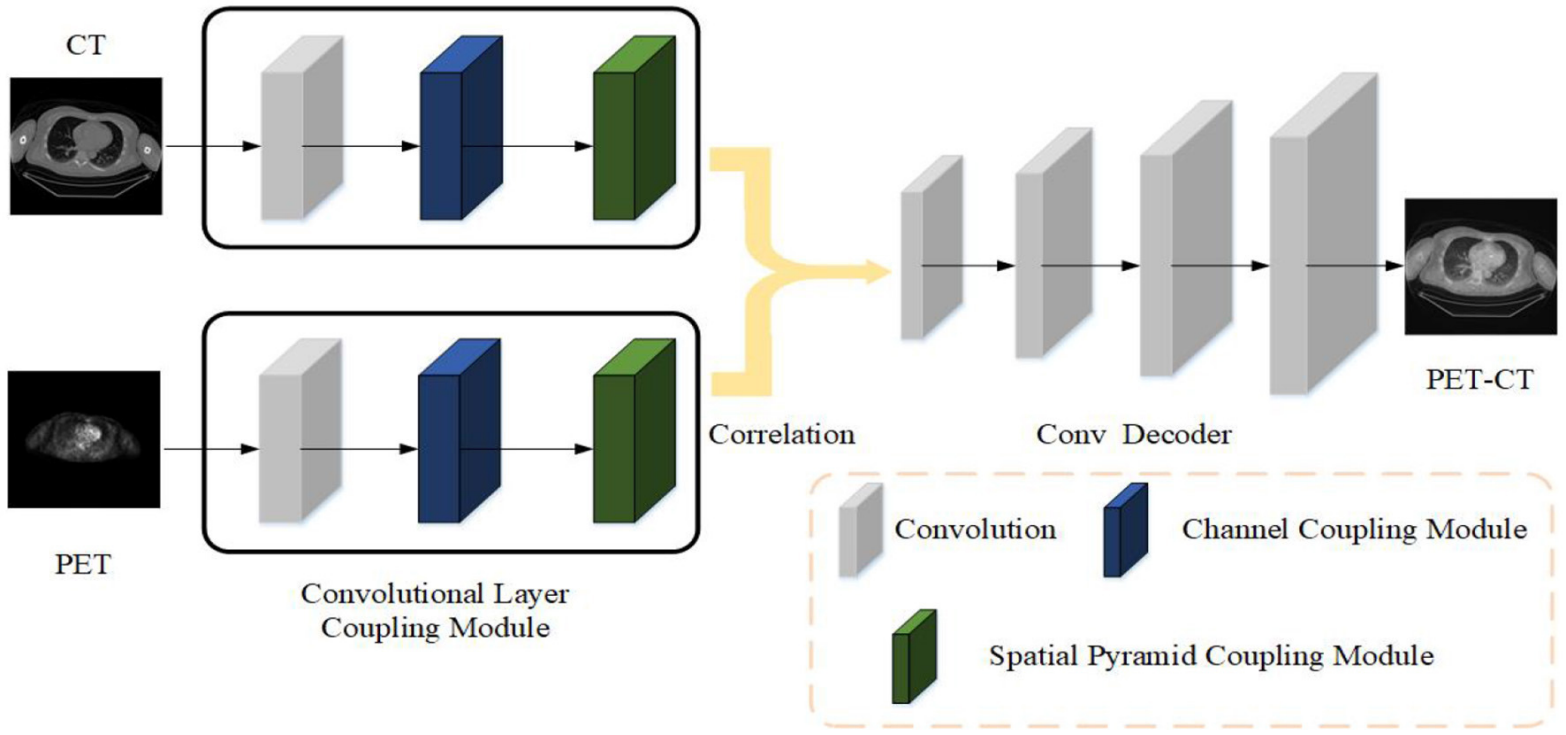
The strategy of the proposed medical image fusion method. The source images first feed into encoder composed of CLCM to extract feature. Then two modality features are fused by cross correlation layer. Finally, the fused PET-CT image is reconstructed through the deconvolutional decoder.

This article uses *X*_*ct*_ and *X*_*pet*_ to represent the original CT image and PET image, *X*_*pc*_ represents the fused PET-CT image, *X*_*pc*_ = *F*(*X*_*ct*_, *X*_*pet*_), *F*() represents the fusion method. In order to be able to find this fusion method, this article designs the Siamese Pyramid Fusion Network (SPFN). When performing image fusion, SPFN is mainly divided into feature extraction part, fusion part, and image reconstruction part. The feature extraction part is mainly composed of two encoders based on the Convolutional Layer Coupling Module (CLCM) to extract features from original image. The fusion part is composed of cross-correlation layer to fuse the characteristics of the two modality images. And the image reconstruction part is a decoder composed of three de-convolutional layers, which decodes the fused features to reconstruct the fused PET-CT image. The specific process is shown in the Figure 1.

### 2.1. Architecture Design

The main framework of the siamese autoencoder architecture proposed in this article is mainly inspired by the siamese network (17). The siamese network initially takes two samples as input and outputs its embedding high-dimensional space representation to compare the similarity of the two samples. Based on this siamese structure, this article designs an improved siamese autoencoder to fuse the features of two samples. The siamese autoencoder includes two encoders, a fusion layer and a reconstruction decoder.

The two encoders have the same structure, sharing parameters, and respectively accept PET and CT as input. In the encoding stage, the two inputs of the siamese encoder are the same size 512 × 512, and the images of the two modalities will be input to the two encoders with different parameters at the same time. The encoder designed in this article is mainly composed of CLCM, which specifically includes three parts: convolutional layer, channel coupling module, and spatial pyramid coupling module.

After the features of PET and CT extracted by the encoder, the features of the two modality images will be fused through the cross-correlation layer, which is implemented by inter-correlation operations. It will be described in detail in Section 2.3. Finally, the fused features are fed into the decoder and reconstructed to obtain the final fused image. The decoder is mainly composed of three deconvolutional layers, which aims to reconstruct the final input according to the features in the latent space. The decoder is mainly composed of three de-convolutional layers, which aims to reconstruct the final input according to the features in the latent space.

### 2.2. Convolutional Layer Coupling Module

In the neural network, data will be transmitted along the designed channel. When a module in the network changes, the data will change accordingly, and it can also affect other channels to change accordingly. Data coupling makes the network model more cohesive, while designing different types of modules can allow the model to dynamically focus on specific types of features in the image, which is more conducive to the PET-CT fusion image of tumor staging.

In this article, we designed the Convolutional Layer Coupling Module. This module mainly includes a convolutional layer, a channel coupling module and a spatial pyramid coupling module. The initial convolutional layer is to extract the feature map from original images. The traditional convolutional encoder is composed of stacked convolutional layers (18, 19). The change in the weight of each layer in this stacked convolutional structure will cause the subsequent output to change accordingly. For fusion tasks, the features extracted by the convolutional layer are different from the heavy classification or segmentation of traditional image processing tasks. There is no need to expand the receptive field to extract features that can distinguish as many categories as possible, but to preserve the details of the image as much as possible. Under the conditions, it is used to characterize the key information in the two modality of PET and CT.

Therefore, after the convolution layer in the encoder, this article does not directly use the stacked convolution operation, but uses the channel coupling module and the spatial pyramid coupling module to extract the multi-scale characterization features of the image. The role of the channel coupling module is to assign different weights for each channel, so that the network can focus on important features and obtain task relevance features. The function of the spatial pyramid coupling module is to transform the spatial information in the original image to another space and generate multi-scale key information (20).

Specifically, this article first uses a set of 3 × 3 size convolution kernels to extract features from the original image. The first layer of convolutional layer can extract some low-level features in the image (21). In the image fusion task, there is no need for the network to process a larger receptive field. In addition, deeper abstract features representing the unknowability of the fusion task will increase the computational complexity of the network. Therefore, after the convolution operation, the stacked convolution and pooling operations are not performed, but the channel coupling module and the spatial pyramid coupling module are used. The two coupling modules perform data coupling. The feature map generated by the convolutional layer determines the input in the channel coupling module. At the same time, the channel coupling module also directly controls the input of the spatial pyramid coupling module.

Given a feature map *F* generated by the convolutional layer, after the coupling module, the fusion task relevance feature map and the multi-scale feature map will be sequentially obtained, and the two types of maps obtained are superimposed with the original image to obtain the final The characteristics of the image. The calculation process of the two types of data coupling is as follows:


(1) $$\begin{array}{r} F^{\prime }=Cc\left(F\right) \end{array}$$

(2) $$\begin{array}{r} F^{\prime \prime }=Cs\left(F^{\prime }\right) \end{array}$$

*Cc* and *Cs* represent channel coupling operation and spatial pyramid coupling operation, respectively. Under the effect of cross-layer connection, the difficulty of training model parameters is greatly reduced, making it easier to train a coding model with good effect. *F*″ is the final output. Next, we will introduce the details of the two coupling module in detail.

#### 2.2.1. Channel Coupling Module

**Figure 2:**
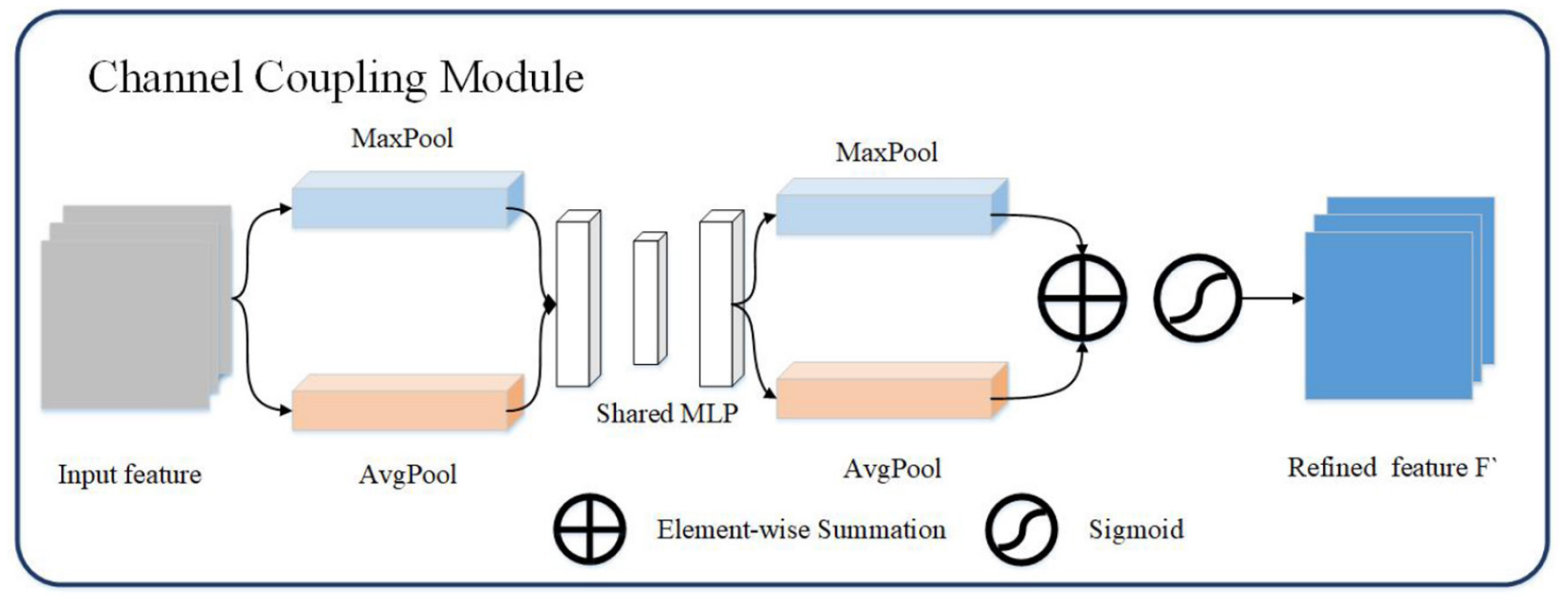
The channel coupling module. The channel coupling module utilizes two different pooling operation and feed results to multi-layer perceptron. The output of multi-layer perceptron continue to forward to element-wise summation and sigmoid operation.

Channel coupling module mainly use the relationship between feature maps to generate channel coupling features. Each channel in the feature map is regarded as a feature extractor, the features extracted by each feature extractor are different, and the focus of the channel coupling module is to find the most meaningful features of the input image in these features (22). In order to effectively extract the channel coupling features of the image, this article designs the structure as shown in the Figure 2. The channel coupling module will pass the input feature map through global max pooling and global average pooling based on width and height, respectively, and then feed into multi-layer perceptron (MLP). The outputs from MLP are performed element-wise summation operation and sigmoid operation. After a series of operations, the final channel feature maps are obtained. The channel feature maps are used as the input of the spatial coupling module. The calculation process for the features of the channel coupling module is as follows:


(3) $$\begin{array}{r} F^{c}=Cc\left(F\right)=Sig\left(MLP\left(AvgPool\left(F\right)\right)+MLP\left(MaxPool\left(F\right)\right)\right) \end{array}$$

*Sig*() indicates that the sigmoid operation is performed on the two sets of results

#### 2.2.2. Spatial Pyramid Coupling Module

Spatial pyramid coupling module mainly uses the spatial relationship between pixels in the image to generate spatial feature maps. Since the convolutional layer only keeps the translation of an object in the image invariance, and the scale transformation of the object cannot be processed, so when extracting the spatial feature map, this article adds the spatial pyramid transformation (23) to extract the multi-scale spatial feature map. The spatial pyramid coupling module is equivalent to performing feature convolution from the bottom up on the feature map of the image, and then fusing feature maps of multiple scales.

**Figure 3:**
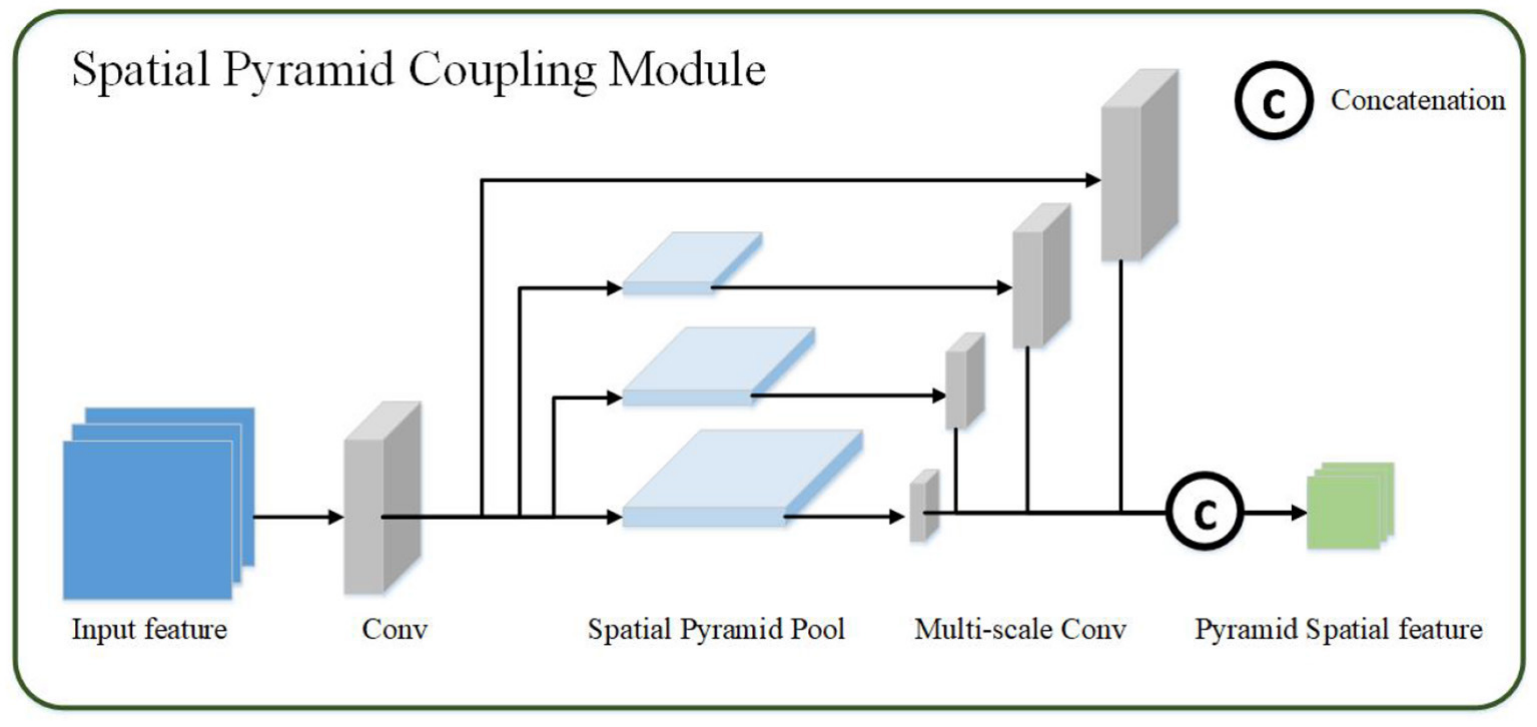
The spatial pyramid coupling module. The spatial pyramid coupling module utilizes spatial pyramid pooling to get multi-scale feature maps and concatenate them.

The spatial pyramid coupling module uses the feature map obtained by the channel coupling module as the input feature map. First, the convolution operation in the spatial pyramid coupling module detaches the input features then uses spatial pyramid pooling to perform multi-scale transformation of the abstract features to obtain four features of different sizes. And the different scales feature maps are obtained through multi-scale convolution after spatial pyramid coupling module. Finally, the four convolution feature results are performed through the channel concatenation operation to obtain the final spatial pyramid feature map, as shown in Figure 3. The calculation process of the spatial pyramid coupling module is as follows:


(4) $$\begin{array}{r} F^{s}=Cs\left(F\right)=Concat\left(Conv\left(SPP\left(Conv\left(F^{c}\right)\right)\right)\right) \end{array}$$

*SSP*() represents for spatial pyramid pooling operation. Specifically, the pyramid pooling operation is to divide the original input features using four different pooling scales (16, 4, 2, 1).

### 2.3. Cross Correlation Layer

For the fusion of PET features and CT features, the simplest fusion method is to linearly add them. However, this operation ignores the association between adjacent pixels in the area, and lacks the expression of the overall information of the image. In order to better enhance the display ability of the fused image features without losing the original information in PET and CT, this article uses the cross correlation layer (24) to fuse the features extracted by the encoder. Given the image features *F*_*ct*_ and *F*_*pet*_, their fusion results will be calculated according to the cross-correlation layer. The specific formula is as follows:


(5) $$\begin{array}{r} F_{pc}=Cor\left(F_{ct},F_{pet}\right) \end{array}$$

*Cor*() represents for cross-correlation layer, and the cross-correlation layer is a special convolutional layer that uses cross-correlation operation. Different from the ordinary convolution function, the cross-correlation function is an operation between two data. When the data is transferred in the network, the weight to be trained is not needed. The calculation equation is as follows


(6) $$\begin{array}{r} Cor\left(x_{1},x_{2}\right)=\underset{m}{\sum }\underset{n}{\sum }x_{1}\left(m,n\right)*x_{2}\left(m+o,n+o\right) \end{array}$$

*x*_1_, *x*_2_ represent the feature patch on the two feature maps *f*_*ct*_ and *f*_*pet*_ respectively, *m*,*n* are the size of patch, *o* represents the patch stride, this article set *o* as 3. Each patch on the feature map *f*_*ct*_ must be cross-correlated with all the patch on the other feature map *f*_*pet*_. In order to obtain the output of the same size as the original image, the padding pattern selects the “SAME” when performing cross-correlation in this article (25, 26).

### 2.4. Loss Function

For the loss function of the network, this article selects the structural similarity loss (6, 27) as loss function. After obtaining the fused PET-CT, it will first calculate the structural similarity with the original PET and CT respectively, as in the formula:


(7) $$\begin{array}{r} SSIM\left(x,y\right)=\frac{2\mu_{x}\mu_{y}+C_{1}}{\mu_{x}^{2}+\mu_{y}^{2}+C_{1}}\cdot \frac{2\sigma_{xy}+C_{2}}{\sigma_{x}^{2}+\sigma_{y}^{2}+C_{2}} \end{array}$$

*x* and *y* are the fused image and the original image, respectively. μ represents image mean, σ_*x*_, σ_*y*_ represent image variance, σ_*xy*_ represents co-variance of images. *C*_1_ and *C*_2_ are constants (avoid the denominator being 0), the calculation formula is $C_{1}=k_{1}L^{2}$,$C_{2}=k_{2}L^{2}$, where *L* is the grayscale change of the image. Since this article is performing feature extraction, the image is normalized, so *L* is 1. *K*_1_ and *K*_2_ are two constants, the default value is 0.01 and 0.03. The loss of the network can be defined as:


(8) $$\begin{array}{r} L_{ssim}\left(x,y\right)=1-SSIM\left(x,y\right) \end{array}$$

When using SSIM, there will be a problem with edge noise defects. Therefore, this article introduces an L1 regularization term (28) in the original loss function. The L1 regularization term ||ω|| can be used to estimate the difference between the target value *x* and the estimated value *y*, which can effectively reduce the noise in the image, has a certain degree of robustness, and can also prevent the neural network from overfitting during training. Therefore, the loss of the final fusion network is as follows:


(9) $$\begin{array}{r} \begin{array}{c} L_{f}\left(x_{ct},x_{pet};y_{pc}|\omega \right)=2-\frac{2\mu_{ct}\mu_{pc}+C_{1}}{\mu_{ct}^{2}+\mu_{pc}^{2}+C_{1}}\cdot \frac{2\sigma_{ct,pc}+C_{2}}{\sigma_{ct}^{2}+\sigma_{pc}^{2}+C_{2}}\\ -\frac{2\mu_{pet}\mu_{pc}+C_{1}}{\mu_{pet}^{2}+\mu_{pc}^{2}+C_{1}}\cdot \frac{2\sigma_{pet,pc}+C_{2}}{\sigma_{pet}^{2}+\sigma_{pc}^{2}+C_{2}}+||\omega || \end{array} \end{array}$$

## 3. Experiment and Result

### 3.1. Dataset

The PET-CT images used in this article are from Soft-tissue-Sarcoma (29). This dataset includes clinical images of 21 patients with lung tumors. All patients underwent FDG-PET and CT screening from November 2004 to November 2011 by McGill University Health Centre (MUHC). The median of intravenous FDG was 420 MBq. The dataset also includes patient information, histopathological type, tumor grade, follow-up information (metastasis, survival rate). In this article, only PET and CT corresponding to the patient's organs including the lungs are used as the experimental dataset, and a total of 840 CTs and corresponding FDG-PET are extracted from the dataset. The PET and CT images of each patient are registered by (30). We adopted a leave-one-out cross-validation strategy to test the effectiveness of the method in this article and divided the data set into a training set and a validation set according to a ratio of 4:1 to 840 pairs of images. In the training verification, 672 imaging data were used as training, and the remaining 168 imaging data were verified, repeated five times. All patient information are de-identified.

### 3.2. Implementation Details

In the used dataset, the resolution of CT is 512 × 512 pixels, and the resolution of PET is 128 × 128 pixels. Before fusion, this article uses image zoom to up-sampling PET to obtain an image with the same resolution as CT. In addition, the human body's absorption of X-rays recorded in CT images, unit is Hounsfield Unit (HU), and the human body's absorption of isotopes recorded in PET images. Therefore, this article uses the min-max standardization method to normalize the images in the extracted dataset. We implement our fusion algorithm using Tensorflow 1.12.0 on a machine running Ubuntu 16.04 with CUDA 8.0 and CuDNN 5.1. Training is performed on 32 GB NVIDIA GTX 1080 Ti. The parameter initialization in the fusion algorithm uses the Xavier method (31); for the optimization algorithm. This article uses the adaptive optimization algorithm AdaGrad algorithm (32) to optimize the parameters.

### 3.3. Evaluation Metric

In order to quantitatively evaluate the performance of the proposed fusion algorithm, there are reference image evaluation indicators and no reference evaluation indicators. This article uses the following seven indicators for evaluation: average value, standard deviation, average gradient, entropy, root mean square error, normalized mutual information, visual fidelity.

The average value $\bar{x}$ of the image represents the average level of the overall pixels of the image and reflects the brightness of the image. Assuming that the size of the image *I* is m*n, the average value of the image is averaged for each pixel,


(10) $$\begin{array}{r} \bar{x}=\frac{\sum_{i=1}^{m}\sum_{j=1}^{n}x_{i,j}}{m*n} \end{array}$$

The standard deviation σ of the image represents the degree of dispersion between the pixel value and the average of the image, and reflects the contrast of the image,


(11) $$\begin{array}{r} \sigma =\sqrt{\frac{\sum_{i}^{m}\sum_{j}^{n}{\left(x_{i,j}-\bar{x}\right)}^{2}}{m*n}} \end{array}$$

The average gradient *G* of the image reflects the clarity and texture changes of the image,


(12) $$\begin{array}{r} G=\frac{1}{m*n}\overset{m}{\underset{i}{\sum }}\overset{n}{\underset{j}{\sum }}\sqrt{\frac{{\left(\frac{\partial I}{\partial x}\right)}^{2}+{\left(\frac{\partial I}{\partial y}\right)}^{2}}{2}} \end{array}$$

$\frac{\partial I}{\partial x}$ represents the gradient in the horizontal direction, $\frac{\partial I}{\partial y}$ represents the gradient in the vertical direction.

The entropy *Ent* of the image is expressed as the average number of bits in the grayscale set of the image, reflecting the spatial characteristics of the grayscale distribution of the image.


(13) $$\begin{array}{r} Ent=-\sum p_{x}lnp_{x} \end{array}$$

*p*_*x*_ represents the proportion of pixels in the image that have a grayscale value of *x*.

The root mean square error *RMSE* of the image is used to measure the difference between the two images. The mean square error is to find the sum of the square of the error for each pixel and find the mean and then square off:


(14) $$\begin{array}{r} RMSE=\sqrt{\frac{1}{m*n}\overset{m*n}{\underset{i}{\sum }}{\left(y_{i}-{\hat{y}}_{i}\right)}^{2}} \end{array}$$

*y*_*i*_ and ${\hat{y}}_{i}$ represent the original image and the fused image, respectively. We calculate the RMSE between the fused image and PET CT, respectively, and take the average value as the final result.

The normalized mutual information *NMI* (33) of the image reflects the information correlation between the two images,


(15) $$\begin{array}{r} NMI=2\left[2+\frac{Ent\left(I_{1},I_{F}\right)}{Ent\left(I_{1}\right)+Ent\left(I_{F}\right)}+\frac{Ent\left(I_{2},I_{F}\right)}{Ent\left(I_{2}\right)+Ent\left(I_{F}\right)}\right] \end{array}$$

where *Ent*(*I*_*k*_, *I*_*F*_) is the joint entropy between the input image *I*_*k*_ and *I*_*F*_.

The visual fidelity *VIF* (34) of the image was originally an evaluation index using the statistical characteristics of natural scenes. The image information will follow the distortion process, which will result in poor visual quality. Therefore, the image quality can be calculated by calculating the fidelity of the image. The visual fidelity of the image can be defined as:


(16) $$\begin{array}{r} VIF=\frac{\sum_{i}I\left(c_{i},f_{i}\right)}{\sum_{i}I\left(c_{i},e_{i}\right)} \end{array}$$

Among them, *I*(*c, f*) and *I*(*c, e*) represent the information that the human eye can extract from the original image and the distorted image, and *c, f, e* represent image blocks of different scales in the image, *i* represents the index of the image block in the image.

In order to verify the quality of the fused images for clinical diagnosis, we also invited four radiologists from the partner hospital to visually evaluate the fused images. Four radiologists evaluated the fusion image from four indicators: Noise Suppression (NS), Artifact Reduction (AR), Detail Information (DI), and Comprehensive Quality (CQ). The evaluation score ranges from 1 to 5 points, with 1 point representing “bad” and 5 points representing “excellent.” Each doctor conducts an independent evaluation.

### 3.4. Comparative Experiment

In order to verify the effectiveness of the proposed method, this article compared five representative methods: Multi-layer Concatenation Fusion Network (MCFNET) (12), Guided Filtering (GF) (9), Adaptive Decomposition(AD) (5), Parameter Adaptive Pulse Coupled Neural Network (PAPCNN) (8), Image Fusion Convolutional Neural Network (IFCNN) (10). The implementation of the above methods has corresponding authors to provide source code, all parameters are the default parameters set by the author. This article verifies from qualitative results, quantitative comparisons, and details of fusion results.

**Figure 4:**
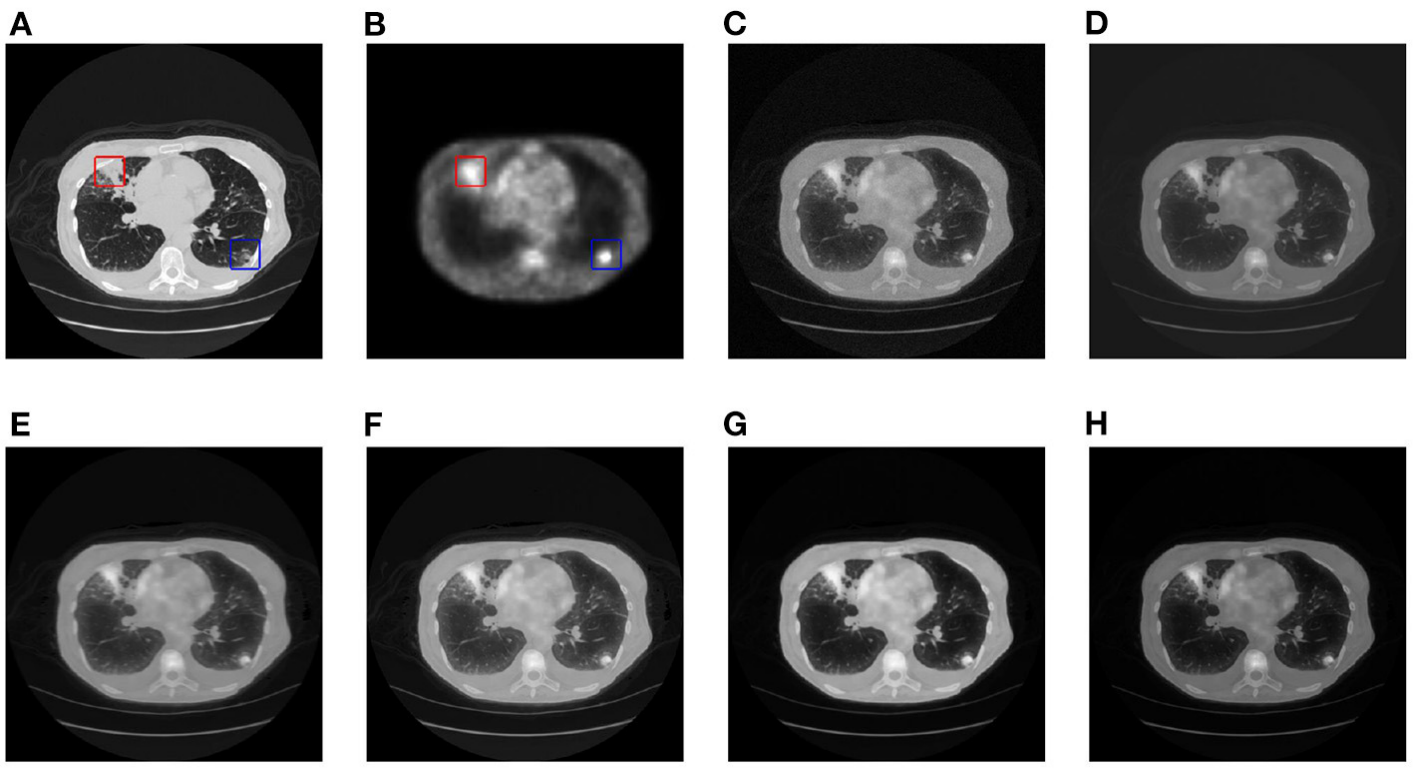
The qualitative comparison results of patient A. **(A)** CT; **(B)** PET; **(C)** AD; **(D)** GF; **(E)** PAPCNN; **(F)** MCFNET; **(G)** IFCNN; **(H)** OURS.

**Figure 5:**
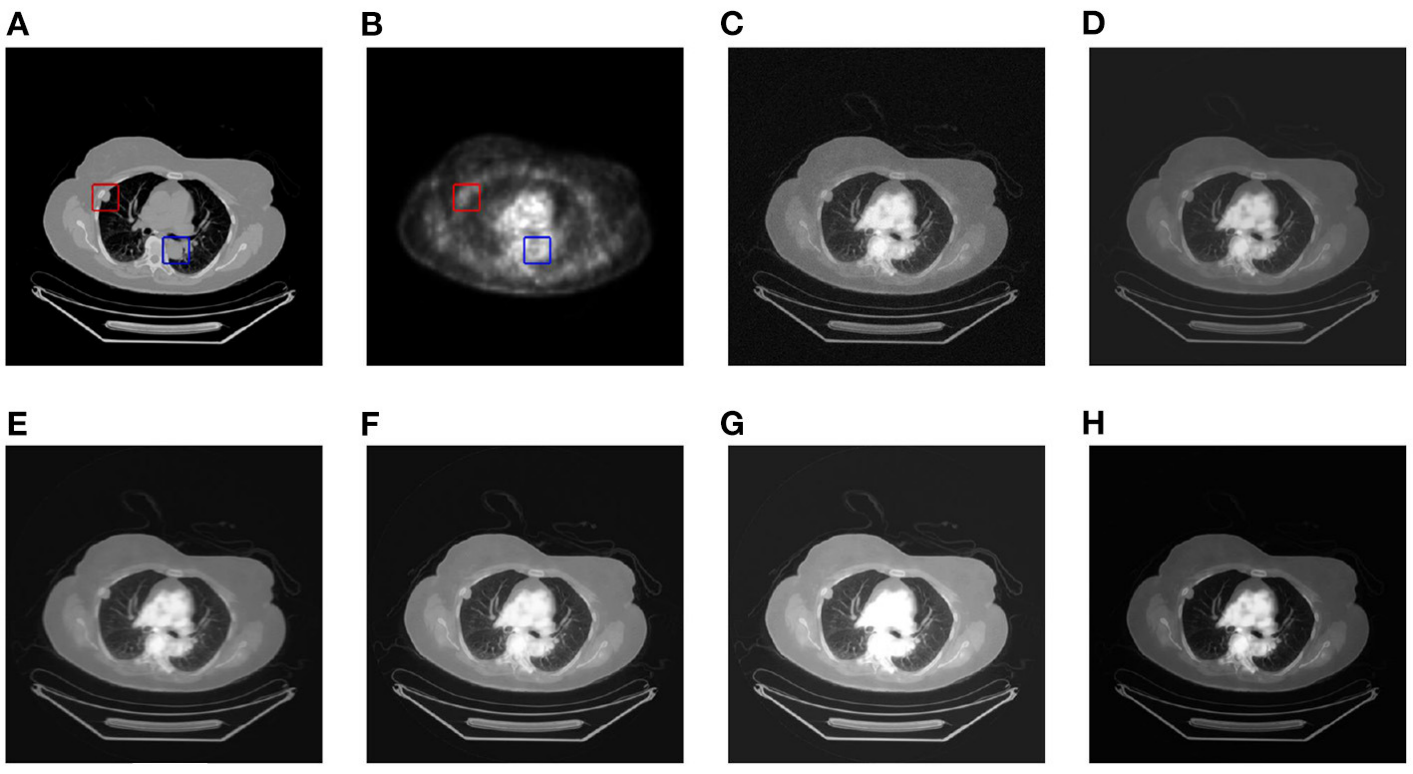
The qualitative comparison results of patient B. **(A)** CT; **(B)** PET; **(C)** AD; **(D)** GF; **(E)** PAPCNN; **(F)** MCFNET; **(G)** IFCNN; **(H)** OURS.

**Figure 6:**
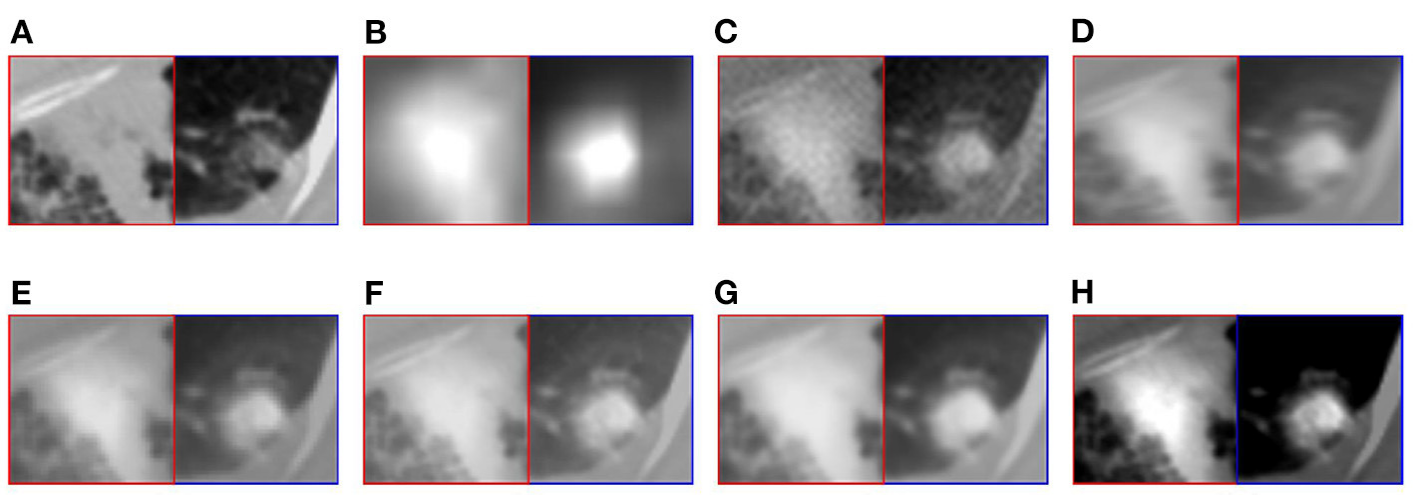
The detail of fusion results of patient A. **(A)** CT; **(B)** PET; **(C)** AD; **(D)** GF; **(E)** PAPCNN; **(F)** MCFNET; **(G)** IFCNN; **(H)** OURS.

This article selects two cases of early lung cancer patients (A and B) from the test dataset for qualitative display, as shown in Figures 4, **6**. It can be seen from Figures 4A, 5A that CT mainly provides detailed edges, contours and other structures of lung lesions and tissues, while PET reflects the accurate location of lung lesions in Figures 4B, 5B. The fusion images of different methods have obtained satisfactory results on the texture and edges, which proves the effectiveness of the fusion method for PET-CT. From the perspective of each fusion result, the fusion image of AD contains some noise in Figures 4C, 5C. In Figures 4D, 5D, the fusion result of GF has low contrast. In Figures 4E, 5E, there are slight artifacts near the lung wall. In Figures 4F,G, 5F,G, the brightness of the fusion result of MCFNET and IFCNN is higher than other results. The contrast and sharpness of the fusion result of the proposed method are relatively high.

**Figure 7:**
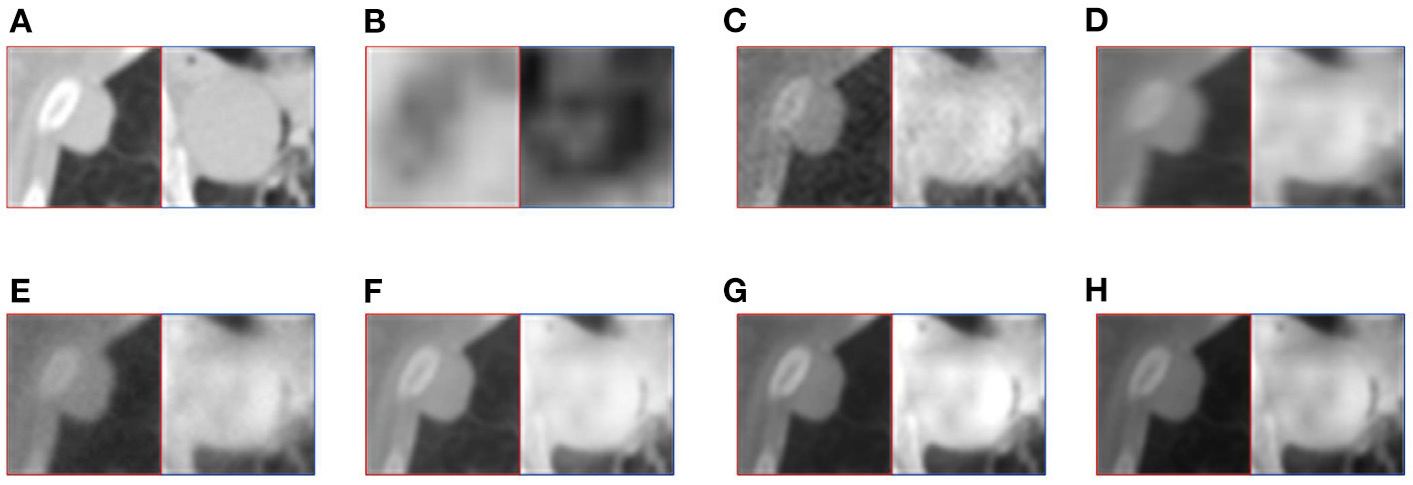
The detail of fusion results of patient B. **(A)** CT; **(B)** PET; **(C)** AD; **(D)** GF; **(E)** PAPCNN; **(F)** MCFNET; **(G)** IFCNN; **(H)** OURS.

For medical images, the brightness of the image is not the main indicator of quality considerations, but mainly the presentation of the lesion area in the image and the display of details. Therefore, this article discusses two details in the original image, as shown in the area marked in Figures 6A,B, 7A,B. The details are shown in Figures 5, 7. We can clearly observe two highlighted areas from Figure 5B, which means that the nodules in this area have rapid metabolism and the nodules are extremely malignant. In the Figure 7, there is an obvious adhesive nodule, however it is not highlighted in PET. This nodule has a slow response rate and low malignancy, which needs follow-up observation. Among these fusion detail results, the fusion results of the proposed algorithm show more excellent results in terms of the contrast between the lesion area and the background and the details of the lesion area.

Next, in order to further verify the effectiveness of the proposed method, this article evaluates the fusion results through different objective indicators and the physician's subjective scores. The specific results are shown in Tables 1, 2.

**Table 1:** Evaluation metric of different fusion algorithm results.

	**Mean**	**Std**	**AG**	**Ent**	**RMSE**	**NMI**	**VIF**
AD	0.102	0.026	0.063	0.051	0.030	3.25	0.321
GF	0.134	0.020	0.072	0.049	0.028	3.16	0.075
PAPCNN	0.204	0.021	0.072	0.065	0.030	3.27	0.303
MCFNET	0.260	0.045	0.091	0.062	0.013	3.25	0.292
IFCNN	0.273	0.065	0.082	0.072	0.019	3.21	0.287
OURS	0.157	0.085	0.091	0.076	0.013	3.28	0.350

**Table 2:** Image quality evaluation scores of different algorithm results.

	**NS**	**AR**	**DI**	**CQ**
AD	2.75 ± 0.43	3.25 ± 0.43	3.75 ± 0.43	3.25 ± 0.43
GF	3.50 ± 0.50	3.00 ± 0.71	3.00 ± 0.71	3.17 ± 0.37
PAPCNN	3.00 ± 0.00	2.50 ± 0.50	3.00 ± 0.00	2.67 ± 0.41
MCFNET	3.75 ± 0.43	3.25 ± 0.43	3.75 ± 0.43	3.58 ± 0.36
IFCNN	4.75 ± 0.43	3.75 ± 0.43	4.75 ± 0.43	4.42 ± 0.28
OURS	4.75 ± 0.43	4.25 ± 0.43	4.50 ± 0.50	4.50 ± 0.37

Table 1 lists the objective results of the entire test dataset for evaluating fusion images of different fusion algorithms. From the results, it can be observed that the standard deviation, average gradient, image entropy, normalized mutual information, and visual fidelity of the original image mentioned in this article have a high level, which proves that the proposed method has a high level of image clarity, contrast and information. The mean value of IFCNN are higher than the method in this article, which reflects that the image fused by IFCNN is better than the method in this article in terms of brightness index, and it also corresponds to the qualitative result.

After displaying the objective evaluation label of image quality, this article also lists the doctor's subjective evaluation scores (*mean*±*std*) of the results of different fusion algorithms, as shown in Table 2.

The evaluation of image quality is one of the main evaluation indicators of the fusion algorithm. In addition, the computational complexity is also one of the factors considered by the fusion algorithm. Therefore, this article also compares the average time for a pair of PET-CT fusion of several algorithms, as shown in Table 3. All fusion methods are run on the same device, and the device parameters are shown in Section 4.2.

**Table 3:** Average runtime comparison of different fusion methods.

	**AD**	**GF**	**PAPCNN**	**MCFNET**	**IFCNN**	**OURS**
Mean time (s)	2.73	1.62	3.38	3.36	3.30	2.12
STD	0.04	0.01	0.20	0.14	0.12	0.03

Among these methods, the fusion speed of the method proposed in this article is slightly slower than that of the GF fusion method at 2.12 s, but it is within the acceptable range, which also indicates that the proposed method has considerable potential in clinical applications.

### 3.5. Results for Staging Diagnosis

One of the main functions of PET-CT is to determine the staging of tumors in patients with lung cancer. In order to evaluate the fusion of PET-CT for the diagnosis of lung cancer staging, this section uses some simple classification methods to evaluate the collected image data and the corresponding staging information. Specifically, different classification methods are used to train and test PET, CT, PET-CT, including Support Vector Classifier (SVC), Multilayer Perceptron (MLP), K-Nearest Neighbor (KNN), Random Forest (RF), and Naive Bayes Classifier (NB), the comparative performance is shown in the Table 4. The kernel function of SVC adopts radial basis function that penalty slack variable is 100 and kernel coefficient is 0.5. The maximum iteration number of MLP is set to 300. The hyper-parameter, neighbor numbers, of KNN is 4. The number of the RF is 100, maximum depth is 2 and the function to measure is gini function. Naive Bayes classifier uses the multinomial Naive Bayes classifier, the number of sample class is 4.

**Table 4:** Classification performance of different modal images for lung cancer staging.

	**SVC(%)**	**MLP(%)**	**KNN(%)**	**RF(%)**	**NB(%)**
PET	62.70	56.66	62.78	60.32	61.80
CT	79.37	79.01	74.86	76.17	81.11
PET-CT	82.71	81.49	80.05	82.34	84.01

As shown in Table 4, both CT and PET have certain staging diagnostic capabilities for lung cancer. However, the performance in staging diagnosis of lung cancer using PET-CT images is generally higher than other monomodal data. The accuracy of constructing individual classifiers in different sample spaces is different, excluding the easy distinguishing characteristics of PET images. PET-CT can improve generalization ability and stability indeed. This result proves that the PET-CT fusion image has diagnostic performance for the staging of lung cancer, which is helpful to assist doctors in identifying lung tumors.

## 4. Discussion

Taking into account the above comprehensive comparison, it can be concluded that the fusion method proposed in this article has a certain competitive performance on the quality improvement and information retention of PET-CT. Nevertheless, this article still has some shortcomings that need to be studied in the future. Since the purpose of this article is to diagnose lung cancer in stages, the collected image data are all CT and PET images of patients, which are limited by technical problems and lack the use of images such as Angiography (35). By selecting different tracers, it can achieve the best results in disease diagnosis. Angiography can show the tumor vascular characteristics of lung cancer and provide a basis for interventional therapy. If the images of different modalities can be fused, the comprehensive judgment of the tumor is of great significance for the stage diagnosis of the tumor and the formulation of the treatment plan.

In addition, the siamese network proposed in this article requires the same resolution of the input two modal data. However, in clinical practice, the resolution of CT and PET are often different. Therefore, in the preprocessing, this article image zoom PET to expand it to the same resolution as CT. Although PET functional imaging does not display too many structure details compared to CT, image distortion often occurs during the up-sampling process. For the fusion of images with different resolutions, how to avoid distortion caused by image scaling is another new challenge.

The main research of this article is the staging diagnosis of lung cancer through PET-CT after fusion. Although experiments have proved that PET-CT after fusion does have a certain improvement in the diagnosis of lung cancer, because of the limited space of this article, only some traditional image classification algorithms are used. In the follow-up work, this article will make corresponding research on the staging diagnosis method of lung cancer.

## 5. Conclusion

In this article, we propose a novel siamese autoencoder network for the fusion of PET and CT. The CLCM in the proposed siamese autoencoder can extract features from both PET and CT modal images, and while ensuring that the original image information remains feature invariance, it can also increase the multi-scale information of the features. In addition, this article also designs a structural similarity loss function combined with the L1 regularization term as the object of the model solution. We collected 840 pairs of PET-CT images to verify the effectiveness of the proposed fusion method in this article. From the results of quantitative comparison, qualitative comparison and subjective evaluation, the performance of fusion results is relatively outstanding, which proves the effectiveness of the proposed method. In future work, we will conduct research on the fusion of PET-CT, aiming to propose high accuracy model for staging diagnosis of lung cancer based on PET-CT.

## Data Availability Statement

Publicly available datasets were analyzed in this study. This data can be found at: https://wiki.cancerimagingarchive.net/display/Public/Soft-tissue-Sarcoma.

## Author Contributions

NX: conceptualization and methodology. WY: visualization. YQ: supervision. JZ and RH: funding acquisition. SL: project administration. JL: data curation and formal analysis. All authors contributed to the article and approved the submitted version.

## Funding

This work was supported by National Natural Science Foundation of China (Grant number 61872261) and Natural Science Foundation of Shanxi Province (201901D111319).

## Conflict of Interest

The authors declare that the research was conducted in the absence of any commercial or financial relationships that could be construed as a potential conflict of interest.

## Publisher's Note

All claims expressed in this article are solely those of the authors and do not necessarily represent those of their affiliated organizations, or those of the publisher, the editors and the reviewers. Any product that may be evaluated in this article, or claim that may be made by its manufacturer, is not guaranteed or endorsed by the publisher.
